# Applying the 3Rs to non-human primate research: Barriers and solutions

**DOI:** 10.1016/j.ddmod.2017.11.001

**Published:** 2017-11-21

**Authors:** Mark J. Prescott, Jan A. Langermans, Ian Ragan

**Affiliations:** 1 National Centre for the Replacement, Refinement and Reduction of Animals in Research (NC3Rs), Gibbs Building, 215 Euston Road, London NWI 2BE, UK; 2 Animal Science Department, Biomedical Primate Research Center, P.O. Box 3306, 2280 GH Rijswijk, The Netherlands; 3 NC3Rs Board, Gibbs Building, 2I5 Euston Road, London NWI 2BE, UK

## Abstract

Progress is being made in the development and application of methods to replace, reduce and refine the use of non-human primates (NHPs) in biomedical research and testing of products and devices. However, there remain considerable cultural and practical barriers to widespread uptake of available 3Rs techniques and to further advancement of the 3Rs in NHP research, over and above scientific obstacles. While most of these barriers apply also to the use of other vertebrate species, there is arguably a greater imperative to overcome them in the case of the NHPs, given their high sentience and the degree of societal concern about their use. To do so will require greater awareness among researchers of the availability and scientific benefits of 3Rs approaches; increased funding for the development of new research models and tools, infrastructure and training; more robust scientific and ethical review of research proposals involving NHPs; better retrospective evaluation of the benefits accrued from NHP research; and improved knowledge transfer. Change is not made without inconvenience, but fully applying the 3Rs to research involving NHPs can improve the quality of science, its translation, business efficiency and public support.

## Introduction

In June 2017, the European Commission’s Scientific Committee on Health, Environmental and Emerging Risks (SCHEER) published a new Opinion on the need for non-human primates (NHPs) in biomedical research, production and testing of products and devices [1]. The SCHEER working group has done what many previous committees reviewing NHP research failed to do — namely conduct a focused, informed and factual analysis of the status of alternatives to NHPs and the available opportunities for applying all three ‘R’s of replacement, reduction and refinement [2]. The importance of this analysis, against a background of polarised debate, rhetoric and misinformation from organisations for and against NHP research, should not be underestimated.

The SCHEER Opinion clearly shows that progress has been made in applying all three ‘Rs’ to different areas of NHP research during the last decade. Most of the advances in science and technology that have contributed to the 3Rs have come from researchers working within their specialist fields, with much of this activity being catalysed by the UK’s national centre for the 3Rs (NC3Rs). Approaches that are contributing to the 3Rs include, for example, greater use of human volunteers, development of non-invasive technologies such as imaging, substitution of NHP models with genetically altered mice, advanced *in vitro* and *in silico* methods, efficient study designs, and application of techniques such as positive reinforcement training to NHP management. The overall message is a positive one, about sustained scientific and medical progress with reduced NHP use and improved welfare.

**Figure 1:**
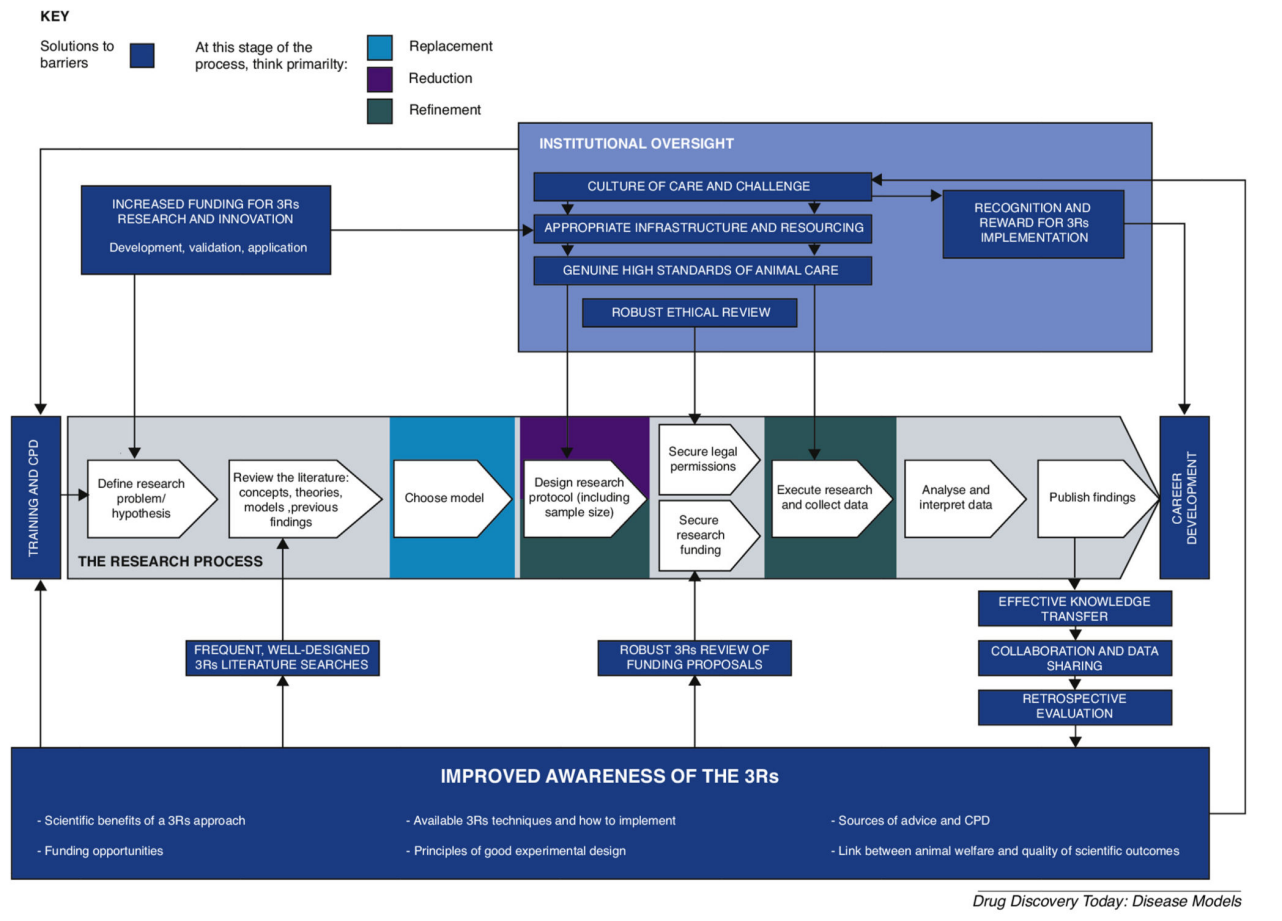
Solutions to the barriers to 3Rs implementation in NHP research.

However, the Opinion also identifies numerous issues that are preventing widespread uptake of these 3Rs advances and further progress. Because 3Rs approaches provide a means of improving scientific quality and translation, failure to address these issues risks not only unnecessary NHP use and suffering, but also poor science and business efficiency [3,4]. Hence the SCHEER working group was motivated to make several recommendations for changes to the status quo. Here we summarise the major barriers to 3Rs implementation and the potential solutions, which are illustrated in Fig. 1. Further information and references are given in the SCHEER Opinion.

### Perceptions of the scientific value of the 3Rs and resistance to change

The 3Rs principles are about change, which can be difficult for humans working in many fields, not just science. Our belief is that while the majority of NHP-using scientists are well intentioned and want to implement the 3Rs, they may encounter several barriers to achieving this objective. All believe their research is important and there is an understandable fear to alter methods that have worked well in the past, or to break away from established models linked to previous funding and the published literature. Pressure in the biomedical research field to publish in high-ranking scientific journals can also force researchers to use established models rather than develop better or new models. Current peer review systems do not encourage change to the extent needed. In addition, NHPs are expensive and only a limited number are available; they are viewed as precious resources, especially once trained, which can inhibit researchers from trying something new that may not work. There can also be a lack of staff, funding and time to develop and validate new approaches which would, if successful, benefit the 3Rs. There is then a need to de-risk new ventures by providing funding for research to try new things and for training and education to support the adoption of novel methods and build confidence in their use.

Progress in 3Rs implementation would be greater if there was wider appreciation of the scientific and business benefits of applying the 3Rs within the NHP research community, and of the link between NHP welfare and quality of science. Poor welfare can cause changes in physiology and behaviour that introduce unwanted variation and confound scientific results [5–7]. As with all models, NHP models have their limitations, despite their similarities with humans. The scientific community needs to more broadly understand these limitations, to select the best model to address a particular question, and accept that sometimes rodent or human-based approaches can be superior, more physiologically relevant or more predictive [8–10]. Scientists knowledgeable about these issues need to do more to raise awareness among their peers. Early career scientists are often more responsive to the 3Rs and new approaches, but it is principal investigators that make decisions in the laboratory and secure the funding. Evidence from other fields suggests that the NHP research community would benefit from more vocal and senior 3Rs advocates. For example, failure to translate promising drug candidates into treatments for asthma has led to questions about the utility of the current animal models and demand for more predictive, human-relevant approaches [11]. Poor translation in stroke research has led to initiatives to improve the quality of experimental design and reporting of *in vivo* rodent studies [12]. In both cases, the support of eminent researchers has been crucial for achieving changes in practice which benefit the 3Rs.

Reluctance to implement available 3Rs techniques can be tackled more directly through changes to the policies and procedures of mainstream funders of biomedical research. For example, to ensure more robust scrutiny of the necessity and justification for NHP use and of the quality of experimental design, some funders now involve experts in alternatives to NHPs, clinicians and statisticians in their peer review processes. Since 2004, the NC3Rs has inputted into the processes of the major UK public funders of NHP research, advising on opportunities to implement the 3Rs in the proposed work and on compliance with guidelines for NHP research that it has developed and the funding bodies have adopted (www.nc3rs.org.uk/integrating-3rs-publicly-funded-research). Where changes are possible to replace, reduce or refine the use of NHPs, these are made a condition of the grant award (regardless of where in the world the work will be conducted) so that they are implemented in practice. Examples of 3Rs impacts achieved as a result of this review are given in the NC3Rs Evaluation Framework [13]. There is scope for other funding bodies to join the NC3Rs peer review and advice service and/or to adapt their procedures to ensure effective implementation of the 3Rs in funded NHP research.

In addition to prospective scrutiny of research proposals for their compliance with the 3Rs, retrospective evaluation of the scientific benefits resulting from the projects, and of any 3Rs impacts, should also be more widely undertaken, in order to make better benefit/harm assessments, which can in turn be used to inform the next generation of research proposals and their assessment by research funding panels [14]. There is also a need for critical appraisal of new technologies which can be positive or negative for the 3Rs. For example, induced pluripotent stem (iPS) cell technology has huge potential for replacement and reduction, but CRISPR/Cas 9 could lead to greater NHP use if scientists seek to develop genetically altered NHP models. Enthusiasm for the potential of new technology should not overshadow the associated ethical and welfare issues, which need serious and timely consideration. Nor should these issues be evaded by transferring such research to regions of the world where lower ethical and welfare standards are accepted.

### Funding for the development and application of alternative approaches

There continue to be research areas for which alternatives to NHPs are not available, and experiments that severely impact the welfare of the NHPs involved (see the SCHEER Opinion). Overcoming the scientific and technological barriers to progress will require research and innovation, but there is a relative lack of funding available. The UK is fortunate in having the dedicated 3Rs funding schemes of the NC3Rs, which has invested over £2 million in research specifically to develop and validate new approaches to reduce and refine NHP use [15]. Aside from this national initiative, funding for 3Rs research in Europe is low. There have been a small number of European Commission funded network activities (e.g. EUPRIM-Net, PRIMTRAIN), but opportunities to conduct 3Rs research tend to be included within much larger calls that focus on other things. Increased funding for the development, validation and application of alternative approaches is critical. The SCHEER recommended a strategic call from the Commission for research aimed at advancing all 3Rs in NHP research. This would help the NHP community meet the policy objectives of Directive 2010/63/EU [16], address public concern about NHP experiments, and validate new alternative or refined methods that have scientific advantages over existing ones.

Funding is also required for improvements in infrastructure, resources and staffing at some NHP facilities. It is ethically questionable to go ahead with NHP experiments if they cannot be performed to the very best standards of welfare and science because of, for example, lack of appropriate imaging facilities, a well-equipped operating theatre, specialist veterinary and animal trainer support, or high quality caging systems. The SCHEER working group recommended that consideration be given to focusing NHP research in centres of excellence. Greater linking of research establishments to share infrastructure, technology and skills could also optimise current use of NHPs and support genuine high standards. Public funders of bioscience research should promote implementation of the 3Rs as an integral part of good research practice. UK funders will consider requests in grant proposals for resources to implement the 3Rs [17]. This is complemented by a specific NC3Rs funding scheme to facilitate the adoption of new 3Rs approaches by transferring knowledge, skills and expertise from the developer laboratory to end-users laboratories.

### Strength of the regulatory framework for NHP use

Complacency about the strength of the European regulatory framework for protecting animals used in science is not matched by the reality. Some claim that Directive 2010/63/ EU “ensures” that NHPs are only used when alternatives do not exist, but this is not accurate and can discourage further efforts to develop alternatives, because it implies that the status quo is sufficient. As pointed out in the Bateson report, NHP use has been justified sometimes on the basis that rodent models are inappropriate when, in fact, the work could have been done in humans; for example, using non-invasive imaging technologies [14]. Nor does the legal requirement to implement the 3Rs always lead to reduction. The number of NHPs used in studies performed to meet regulatory requirements can be highly variable and is not always based on scientific criteria [18]. Even in the academic sector, NHP numbers are not always well justified and experimental design and reporting are not exemplary, contributing to problems with reproducibility and/or translatability of results to humans [19,20] — hence the recent focus on this from some funding bodies [21]. Research publications reveal that licensed research is not always fully refined, leading to unnecessary suffering. There can be many reasons for this, including a lack of awareness of existing refinement opportunities and their relevance on the part of individual researchers and those involved with reviewing their work during the ethical review and approval process.

We fully support the need for legal controls and ethical review of NHP projects, but there is also a need to inspire change based on the scientific benefits the 3Rs can bring, aside from the regulatory framework which can be perceived as anti-science and bureaucratic. The NC3Rs has demonstrated that influencing from within the scientific community, whilst simultaneously providing the support needed for making change (e.g. funding for research and infrastructure, networks and platforms for data sharing, web-based training resources), is a strategy for change that is complementary to regulation and arguably more effective. Individual researchers and organisations have responded positively to its science-led and collaborative approach. There is no reason to suspect that similar advances could not be made in other countries if they adopted a similar model.

Around 75% of the NHPs involved in scientific procedures are used for non-clinical safety testing of new chemical and biological products and devices [1]. Whilst this testing is a regulatory requirement before initiating clinical trials in humans, none of the regulatory guidelines give clear recommendations on study design, in particular in relation to the number of animals used, and there is scope in the choice of species. Although intended to allow flexibility in approaches for individual programmes, this ambiguity can lead to different perceptions of the regulatory requirements, resulting in large variation in the number of animals used for similar studies. Since NHPs are often the only pharmacologically relevant species for the majority of antibody-based products, increased NHP use is anticipated as the market for biotherapeutics rapidly expands. Finding ways to stem this increase is therefore critical. By acting as an honest broker for data sharing between pharmaceutical companies and regulatory bodies worldwide, the NC3Rs has identified many opportunities to reduce, and in some cases avoid, NHP use, without compromising human safety [22]. For example, the number of NHPs required per monoclonal antibody in development can be reduced by up to 64% via the use of efficient study designs, which are being implemented by the companies involved. This NC3Rs-industry work illustrates the need for case-by-case science-driven decisions on the necessity for NHP data and use of published efficient study designs, as well as the benefits of companies and regulators working together to influence change. The savings from this approach, in terms of animals, cost and time, can be significant.

### Awareness raising, training and knowledge transfer

3Rs opportunities are not universally applied, despite the regulatory requirement to do so, because some researchers, animal care staff and members of ethics committees lack awareness about them. Oversight and enforcement can be lax, as shown by recent exposés of poor practice that existed despite there being formal structures in place in compliance with the legal requirements. Laboratory personnel are busy people, but a failure to identify and exploit the latest 3Rs-relevant methods contravenes the terms under which permission is granted for invasive NHP research and risks poor or outdated science. All should take seriously the responsibility to conduct well-designed literature searches at the outset of their projects and at regular intervals afterwards to keep abreast of published 3Rs-related research. Responsibility for horizon scanning for 3Rs advances can be delegated to a nominated person within the laboratory. There is also a need for greater networking and exchange visits between research groups using NHPs, to share information on how to successfully apply the evidence base and new 3Rs techniques. Mainstream conferences and dedicated events such as the annual NC3Rs Primate Welfare Meeting provide a platform for this within Europe. A recent publicly funded initiative in France is seeking to standardise practice and disseminate technological advances in line with the 3Rs across national NHP laboratories through mailing lists and an annual meeting (http://biosimia2017.sciencesconf.org/).

Organisations which represent NHP researchers also need to step up and play a proactive role in raising awareness about 3Rs opportunities among their membership and in the public domain. In this context, it is extremely disappointing that there has been almost no coverage of the final SCHEER Opinion by learned societies, industry bodies and lobby groups connected with NHP use. This contrasts sharply with the rush to defend the status quo, and to play lip service to the 3Rs, when faced with opposition from antivivisectionists or allegations of malpractice. Some of the SCHEER conclusions may be difficult for the community to handle (e.g. that alternatives exist for some areas of NHP research, that available 3Rs techniques are not universally applied, that some NHPs experience severe suffering), but failure to properly engage with the issues risks organisations being out of step with the scientific reality and the more enlightened attitudes of individual members.

Scientific knowledge relevant to the 3Rs can be underused due to poor reporting and dissemination. Researchers need to use the ARRIVE Guidelines (www.nc3rs.org.uk/ARRIVE) when reporting NHP studies in the literature and make their data sets available for others to use. NHP research is sometimes justified by the need to cure human disease without good prior evidence or retrospective analysis of medical benefits/translation. Overstating the case for NHP research makes it an easy target for organisations which oppose animal use. Better reporting and greater data sharing would enable systematic reviews and meta-analyses to address this issue, enabling efforts to be focused on the most valuable research models and areas. Publication of null and negative findings would help to avoid unnecessary duplication of studies.

There is also room for improvement in the provision and quality of staff training and the research culture at some NHP research establishments. All those involved with NHP research should have initial training which provides a strong foundation in the 3Rs and relevant techniques, including state-of-the-art methods, and have good support for continuing professional development throughout their careers. Greater use should be made of the FELASA-accredited courses established under EUPRIM-Net and the online resources from the NC3Rs (e.g. Macaque Website, Common Marmoset Care, Experimental Design Assistant; www.n3rs.org.uk/resources). Institutional oversight mechanisms should support best practice via a culture of care, with recognition and reward for commitment to the 3Rs, for example through job promotion, letters of commendation or prizes. A culture of challenge (e.g. of poor or outdated practices and attitudes) is similarly important. It should not be acceptable for staff to “hide behind” the legislation, implying that they are following best practice merely on account of their research having been licensed by the competent authority. Sanctions and compulsory retraining have been necessary in some cases.

## Conclusions

The SCHEER Opinion was commissioned in advance of this year’s review of Directive 2010/63/EU [16]. Whether the European Commission and NHP research community will act on the recommendations from the SCHEER remains to be seen. There is opportunity for the Opinion to mark a watershed moment — the point at which the entire community, in its widest sense, became serious about the 3Rs. We believe NHP researchers and others are open to change if they can see the benefits it will bring to their science and careers. With the right combination of encouragement, funding, information dissemination and support, many more could become involved in delivering 3Rs impact, using the most relevant and predictive tools and models. This would not be at odds with scientific and medical progress and could, in fact, accelerate it. Campaign groups have repeatedly called for a ban on NHP experiments, and claim this would catalyse greater activity to develop alternatives. Rather than risk the impact a ban could have on advancing scientific knowledge and human health, it is far better for the NHP community to be proactive and at the vanguard of a science-led approach to replacing, reducing and refining NHP use. This will be particularly important given potential drivers for increased NHP use in the near future such as new genome editing technologies and an increasing volume of biologics in the drug development pipeline.
